# Pilot Study of the Influence of Equine Assisted Therapy on Physiological and Behavioral Parameters Related to Welfare of Horses and Patients

**DOI:** 10.3390/ani11123527

**Published:** 2021-12-10

**Authors:** María Dolores Ayala, Andrea Carrillo, Pilar Iniesta, Pedro Ferrer

**Affiliations:** 1 Department of Anatomy and Comparative Pathological Anatomy, Faculty of Veterinary, Campus of Espinardo, University of Murcia, 30100 Murcia, Spain; andrea.carrillo1@um.es; 2 Centauro-Quirón Foundation, Camino Salitre, 28, Junto al Río Mula, Alguazas, 30560 Murcia, Spain; pilicainiesta@gmail.com (P.I.); p.f.ferrer@hotmail.com (P.F.)

**Keywords:** equine assisted therapy, horse-patient interaction, stress, well-being, quality of life

## Abstract

**Simple Summary:**

Equine assisted therapy is being used successfully in patients with different psychic and motor pathologies. In the present work, different physiological and behavioral parameters have been evaluated in horses and patients with psychomotor alterations during equine assisted therapy sessions. After a first anticipatory phase in which signs of stress were observed in the behavior of the horses, with increased heart and respiratory rates as well as increased blood pressure, the horses relaxed in the phase of interaction with the patients on the ground. Later, in the phase of interaction with the patient on horseback, physical activity increased the heart and respiratory rates in the horses, but these parameters decreased after finishing the sessions (recovery phase). The patients also showed some anticipatory stress with increased heart rate in the first phase, but they relaxed during the interaction phases on the ground and on horseback. In addition, the quality of sleep improved on the days of therapy. On the other hand, the patients improved their fine and gross motor function, as well as the parameters related to the cognitive, emotional and affective-social areas. The benefits in the patients had a positive influence on the quality of life of their families.

**Abstract:**

Different welfare indicators were studied in three patients with psychomotor alterations and in two horses throughout 9–10 equine assisted therapy sessions in each patient. In horses, heart and respiratory rates, blood pressure, temperature and behavioral signs were studied. In patients, heart rate, oxygen saturation, temperature, sleep quality, psychomotor and emotional parameters were analyzed. Data collection was recorded in the anticipatory phase (15 min before the start of the session), two interaction phases (after 30 min of horse-patient interaction on the ground and on horseback, respectively) and the recovery phase (15 min after the end of the session). During the anticipatory phase, most of physiological parameters of patients and horses and the stress behavioral signs of horses increased, followed by a relaxing phase during the horse-patient interaction on the ground. In horse-patient riding phase the heart and respiratory rates of the horses again increased. These results showed that the horses did not seem to suffer stress attributable to the therapy sessions, but only an increase in their parameters associated with activity and external stimuli. The patients improved their gross and fine motor skills, their cognitive and perceptual-sensitive parameters and it led to an improvement in the life quality of their families.

## 1. Introduction

So far the studies carried out on Animal Assisted Interventions (AAI) have been mainly focused on the well-being of the patients. In general, these studies show a positive effect of equine assisted therapy in patients with cerebral palsy and other psychomotor disorders, with an improvement in global motor development [1,2,3,4,5,6,7]. Thus, studies carried out on equine assisted therapy show an improvement in balance, range of movements, postural control, functionality and motor control [4,6,8,9,10]; as well as an improvement in cognitive, sensory and emotional capacities [3,5]. Some authors [11] determined that the functional improvements obtained in children with infantile cerebral palsy were, essentially, due to the movements the horse transmitted to the rider’s body, these impulses being three-dimensional, rhythmic and equivalent to the physiological pattern of human walking. According to the published scientific literature, the benefits of the equine assisted therapy are many and no negative effects have been observed in patients who have been treated with these therapies. However, it is not possible to generalize the results that have been published so far, due to many factors such as small sample sizes, heterogeneity in terms of age or clinical manifestations [4]. On the other hand, the International Association of Human Animal Interaction Organizations IAHAIO establishes a series of guidelines that must be taken into account in AAI to avoid harm to people and therapy animals (co-therapist). Thus, patients should not suffer any specific allergy to the breed or species of animal used during the session and an exclusion criterion should be implemented for “high risk” cases (immunosuppressed patients or patients with diseases transmitted through animals). In addition, equine assisted therapy is contraindicated in cases where the patient should not be mobilized and where inflammatory processes are present. Regarding animal welfare, according to IAHAIO guidelines, the AAI sessions should only be carried out with the help of animals that are in full health, both physically and emotionally, and that enjoy this type of activity. It is imperative that the animal handler is familiar with the participating animal in the intervention. It is the responsibility of professionals to ensure the welfare of the therapy animals and to consider the well-being and safety of all participants. Professionals must understand that the participating animal, regardless of the species, is not simply a tool, but living being.

Currently there is a growing number of works aimed at studying the welfare of the therapy animal that include, among others, studies on physiological, behavioral and hormonal parameters. Previous authors [12] studied the behavior of horses in therapeutic riding programs. No significant differences in stress-related behaviors were found by these authors when horses were ridden by recreational riders, physically and psychologically handicapped riders or special education children. However, the mean number of stress-related behaviors was significantly higher when horses were ridden by the at-risk children (children of low socioeconomic status, poor school performance or disciplinary action). According to these results, the authors suggested that for horses in a therapeutic riding program, being ridden by physically or psychologically handicapped individuals is no more stressful for the horses than is being ridden in the same setting by recreational riders. However, since that at-risk children caused more stress to the horses, the authors suggested that the time horses are ridden by at-risk children should be limited both daily and weekly. On the other hand, an ethogram was developed in the cited study [12], that included specific equine behaviors that indicated stress, irritation, or frustration on the basis of results of previous studies [13,14,15,16] and the experience of the investigators. The ethogram developed in the cited study is currently used as a valid reference in stress studies of horses used in assisted therapy. For this reason, this ethogram has also been used in the present work (see Section 2).

Likewise, other authors [17] compared the level of salivary cortisol in horses that were used in therapeutic riding with the cortisol released by the same horses in non-therapeutic riding sessions. Additionally, the horses of that study were scored for different stress behaviors based on the ethogram developed by the authors previously cited [12]. Salivary cortisol concentrations were not higher in horses during therapeutic riding, compared to hunt seat riding or rest conditions. Similarly, behavioral evaluations of the horses during these different activities did not reveal differences in stress levels. According to these findings, the authors suggested that therapeutic horseback riding did not induce more stress than hunt seat riding for the horses involved.

Other works also include the study of these parameters simultaneously in co-therapist horses and in the patients that interact with them in AAI sessions, in order to observe the effect of the interaction between both (horses and patients). Thus, some authors measured different physiological, hormonal and behavioral parameters in co-therapist horses and patients and obtained values similar to those obtained in horses that interacted with healthy people [18,19,20]. Thus, some authors [18] exposed horses individually to pairs of humans who physically resembled each other and moved in a similar way, however one of those humans suffered from Post-Traumatic Stress Disorder (PTSD) while the other did not. Physiological and behavioral measurements showed that horses were less stressed when any human was present with them, and more attentive toward humans who were more experienced with horses. Additionally, horses appeared to respond more to physical cues from the human rather than implied emotional needs. Additionally, it was clear that the presence of any human was preferred by the horse to being alone. Horses alone in the round pen displayed a variety of stress behaviors including increased vocalizations and faster gaits, however when any human joined them regardless of her mental state, stress levels decreased as evidenced by horses [18]. Other authors [19] assessed levels of stress and or well-being in horses involved in equine assisted therapy, as measured by plasma cortisol and oxytocin and by heart rate, and assessed the effect of equine assisted therapy on several physiological measurements and change in symptoms of PTSD in veterans. The participants reported a significant decrease in symptoms of anxiety and depression along with other symptoms of psychological distress and PTSD. Additionally, heart rate decreased specifically on the day when the veterans were more stationary and were primarily engaged in grooming and petting the horses, versus actively leading and walking around. Stress levels were not observed in horses, as demonstrated by plasma cortisol concentrations and heart rate. Furthermore, the horses did not demonstrate increased levels of well-being as demonstrated by the lack of change in plasma oxytocin concentrations after equine assisted therapy sessions. Other authors [20] studied the impact of equine-assisted therapy on equine behavioral and physiological responses, such as heart rate. The results suggested that horses experienced neither negative nor positive emotions during equine assisted therapy sessions. In addition, horses rapidly balanced their autonomic nervous system after these sessions. Hence, the authors suggested that the horses involved in these therapy sessions perceived the activities as neutral rather than negative or positive.

The present work is aimed at evaluating the influence of the equine assisted therapy sessions on the well-being of horses that are dedicated to equine assisted therapy and relating the results with those obtained in patients and their families. To do this, we have analyzed different physiological and behavioral parameters in horses and patients during equine assisted therapy sessions and we have evaluated the influence of this therapy on the quality of life of the patients and their families. This work has been carried out in collaboration with the Centauro-Quirón Foundation, which is a Spanish institution dedicated to animal assisted therapy.

## 2. Material and Methods

### 2.1. Facilities and Characteristics of the Assisted Therapy Horses and Patients

This work has been carried out at the facilities of the Centauro-Quirón Foundation (C-Q, Murcia, Spain. REGA ES300305440012), dedicated to AAI. Three patients and two assisted therapy horses from C-Q Foundation were studied. Table 1 and Table 2 show the characteristics of the horses and of the patients, respectively. The morphometric measurements of the horses were made according to other published studies [21]. The objectives of the equine assisted therapy sessions were to improve the motor functionality through exercises on the ground and on horseback riding; and to enhance the psychological, psychic and emotional aspects through the contact and the bond of the patients with the horses. The team that took part in the sessions was made up of an occupational therapist, a physiotherapist, a speech therapist, a psychologist and a horse trainer-guide.

The present work complies with the Guidelines of the European Union Council (2010/63/EU) and was approved by the Animal Experimentation Ethics Committee and by the Human Research Ethics Commission of the University of Murcia (Murcia, Spain).

### 2.2. General Structure of the Equine Assisted Therapy Sessions and Data Collection

To carry out this study, each patient attended 1 session per week of equine assisted therapy for 9–10 weeks. Each session lasted ≈60 min: during the first 30 min the patient interacted with the horse on the ground (HP ground phase) and during the next 30 min the patient performed a horseback ride (HP riding phase). For the present work, the back riding technique (passive equine assisted therapy) was carried out. The horses were only ridden at the walk in the present study. The Table 3 explains the characteristics of the phases that were analyzed in each session as well as the times when data collection took place. Control measurements were also performed (control phase): the same parameters as in the therapy phases were recorded weekly at the same time of day as the therapy sessions, but 2–3 days away from the therapy sessions, on rest days for horses and patients. Furthermore the patients’ sleep quality was monitored on the days of equine assisted therapy (sleeping phase) and on control days (control phase).

### 2.3. Physiological Parameters of the Horses

The temperature (T) was recorded about 10 cm away from the anus area, using an Aposán brand infrared thermometer (model JXB-190) (Wellkang Ltd., Dublin, Ireland). The heart rate (HR) was obtained by palpation of the facial artery, near the incisura of the facial vessels. The respiratory rate (R) was obtained by observing the movements of the rib cage. Inspiration and expiration were counted as one breath. The blood pressure (P) (systolic SBP/diastolic DBP) was monitored with a Quirumed brand blood pressure monitor (model QMTEN5-75) (Tianjin, China), located at the base of the tail in contact with the middle caudal artery. The measurements of each parameter took few minutes since they were repeated several times to obtain an average value. Reference rectal temperature values in horses are between 36 and 38 °C [22]. Heart and respiratory rates vary with race, age, size, etc. However, according to the consulted bibliography, the reference values in an adult horse are 25–40 beats per minute at rest [23]. A trained horse will have lower values. The respiratory rate also depends on many factors. However, the averages of respiratory rate at rest during the morning are 24.8 ± 3.5 breaths per minute [24]. The physiological values of systolic blood pressure (SBP) and diastolic blood pressure (DBP) in horses are 116.54 ± 8.97 mm and 64.74 ± 12.94 mm Hg [25], respectively.

### 2.4. Behavioral Parameters of the Horses

The signs of pain and/or distress were collected in the horses according to the animal experimentation protocols of the Spanish committees of ethic and of animal experimentation of the University of Murcia (Murcia, Spain), as well as according to those published by previous authors [12]. The cited researchers [12] studied the signs of stress in horses used in therapeutic riding. According to their results, these authors developed a specific ethogram that is currently used by researchers as a valid reference for the study of behavioral signs of stress in equine assisted therapy. Hence, the ethogram published by these authors was also used in the present study. The measurements of the stress behavior were carried out as follows: every 4–7 min (depending on the duration of each phase), the signs of stress were recorded. To do this, a person was exclusively dedicated to visualizing these signs and writing them down. The percentage of appearance of each sign was calculated at the end of each phase from the results that were observed.

### 2.5. Physiological Parameters of the Patients

The T was measured with the infrared thermometer previously described for the horses, and it was taken at about 5 cm away from the temporal lobe. The heart rate (HR) and oxygen saturation (O) was measured with a Choice MMed’s children pulse oximeter (Beijing Choice Electronic Technology Co., Ltd., Beijing, China). On the other hand, a Xiaomi fitness wristwatch (“Mi Band 4” model) (Anhui Huami Information Technology Co., Ltd. Hefei, Anhui, China) was used to measure the HR of the patients at home and to obtain the controls measurements. Additionally, the wristwatch was used to analyze the quality of sleep: it recorded the total hours of sleep, differentiating between the hours of light sleep and those of deep sleep. These data were recorded on the nights that the patients had received equine assisted therapy (sleeping phase) and in control days, that were collected on nights away from the therapies (control phase).

### 2.6. Motor, Cognitive and Perceptual-Sensitive Parameters of the Patients

At the beginning of the experiment, the therapeutic team of the C-Q Foundation carried out an evaluation of the motor, cognitive and perceptual-sensitive aspects in each patient using a list of parameters that is shown in the Table S1. The therapeutic team used these parameters, taking as references those used by other authors [26]. However, these parameters were adapted by the C-Q team according to the characteristics of their patients and the specific criteria of the therapists. At the end of the experiment, the same evaluation was again performed in each patient to detect the effects of the equine assisted therapy throughout the 9–10 sessions. However, since only three patients were studied, and their characteristics (age, pathology, etc.) were heterogeneous, the statistical analysis and calculation of mean values of these parameters were not possible. The therapeutic team only collected a general assessment of the changes observed in the three patients to assess whether the sessions had been beneficial or not. Therefore, these data only represent an indicative value of the potential of these therapies and show the need to carry out studies with a greater number of patients and higher homogeneity of their characteristics in order to objectify the results.

### 2.7. Quality of Life Parameters of the Patients and of the Families

At the end of the 9–10 sessions of equine assisted therapy in each patient, some surveys (of our own elaboration) were given to the families –to collect the assessment of the families in relation to the perceived changes in the patients and in the families after the sessions. The surveys were structured in a very easy way so that families were able to answer objectively and numerically. The aspects to value were listed in the surveys and each one of them was rated from 0 (0% perceived improvement) to 5 (100% perceived improvement). Thus, the data are expressed as a percentage of improvement according to the family members’ score. Additionally, the families could add any other aspect (positive or negative) that they had detected in relation to the therapies. However, the families did not detect any negative effects, as reflected in the surveys.

### 2.8. Statistical Analysis

The statistical analysis was performed with Statistical Package SPSS 24. The mean and standard error of the mean (SEM) were calculated for each parameter in each phase in horses and in patients. To do this, every week we collected data in all phases of each weekly session. At the end of the 9–10 weeks of study, we combined the data from each phase and compared them with the other phases, in horses and in patients. The statistical analysis was performed to determine if there were differences among the different phases for each parameter. In addition, we did a second statistical analysis to observe if the parameters of each phase changed throughout the weeks in order to determine if the number of sessions influenced the physiological and behavioral parameters. The data distribution and the homogeneity of variances were analyzed by the Shapiro-Wilk and the Levene’s tests, respectively, for *p* < 0.05. For most of the parameters, both tests showed values of *p* > 0.05 and hence, the analysis of variance (ANOVA) and a post-hoc Tuckey test were used, for *p* < 0.05. However, nonparametric tests (U of Mann-Whitney and Z of Kolmogorov-Smirnov tests) were used in the cases with values of *p* < 0.05.

## 3. Results

### 3.1. Physiological and Behavioral Parameters in Horses and in Patients during the Equine Assisted Therapy Sessions

**Figure 1:**
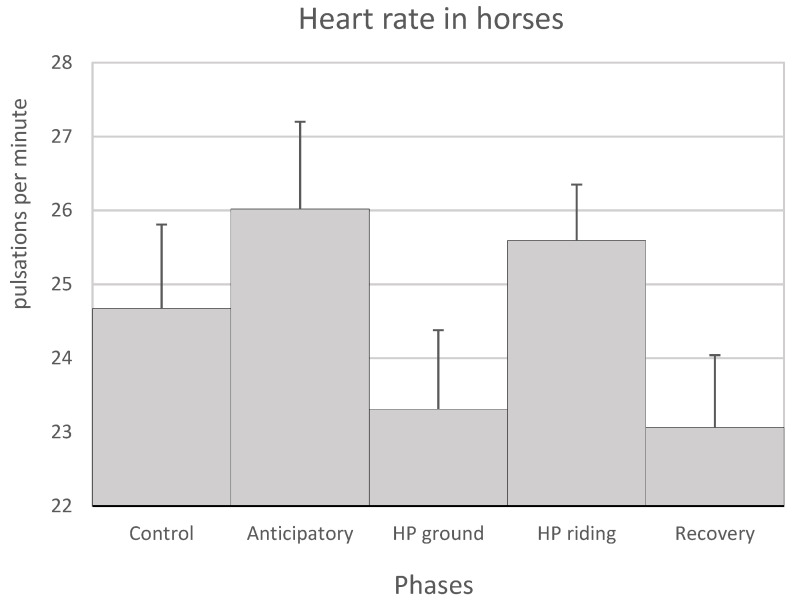
Mean values + SEM of the heart rate of the horses in the control and the anticipatory phases, the HP ground and HP riding phases (horse-patient interaction on the ground and on horseback, respectively) and the recovery phase. No significant differences were observed between the phases.

**Figure 2:**
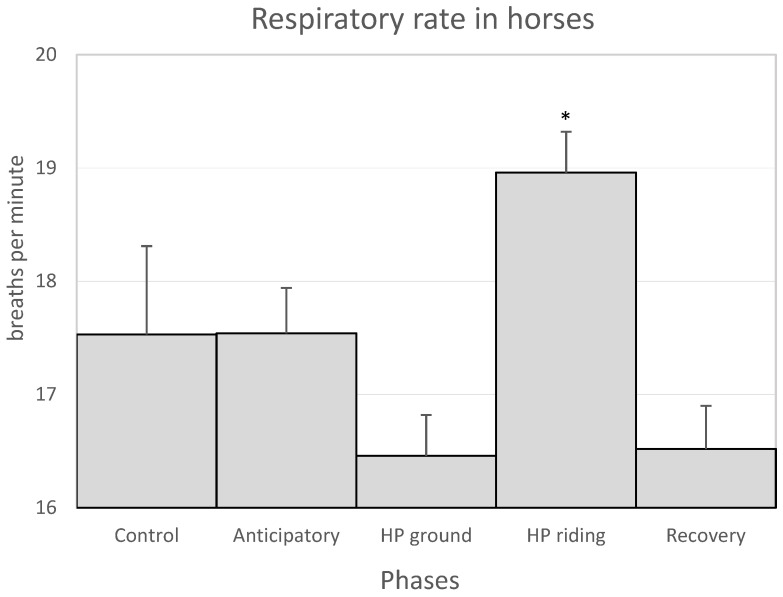
Mean values + SEM of the respiratory rate of the horses in the control and the anticipatory phases, the HP ground and HP riding phases (horse-patient interaction on the ground and on horseback, respectively) and the recovery phase. The asterisk indicates that the values were significantly higher in the HP riding than in the HP ground and recovery phases (*p* < 0.05).

The physiological parameters that were measured in the horses and in the patients remained within the normal range of each species throughout the sessions. As described in the Section 2, the following phases were studied in each session: a first phase –or anticipatory phase, a second phase –or horse-patient interaction phase on the ground (HP ground phase), a third phase or horse-patient interaction on horseback (HP riding phase) and a fourth or recovery phase (Table 3). In the horses, the highest HR and R values were observed in the anticipatory and HP riding phases, whereas the lowest values were observed in the HP ground and recovery phases. However, the HR values did not show significant differences among the different phases, while the R was significantly higher in the HP riding phase than in the other phases (Figure 1 and Figure 2). For its part, blood pressure gradually decreased from the anticipatory phase to the recovery phase, in which their values were similar to the control values (Table 4). The T was similar in all the therapy phases (Table 4). On the one hand, the physiological parameters of each phase were compared throughout the 9–10 sessions to study whether the number of sessions could influence them. The study showed that the HR values of the anticipatory phase gradually decreased throughout the sessions. However, these results are not significant (*p* > 0.05) (not shown).

In relation to the behavioral signs of stress in horses, a higher frequency of them was observed in the anticipatory phase (Table 5) with a tendency to decrease in the subsequent phases, even though the response was not homogeneous in all signs. When observing the evolution of these parameters throughout the 9–10 sessions, a tendency to decrease the frequency of these signs was observed, especially in the anticipatory and recovery phases (*p* > 0.05) (not shown).

**Figure 3:**
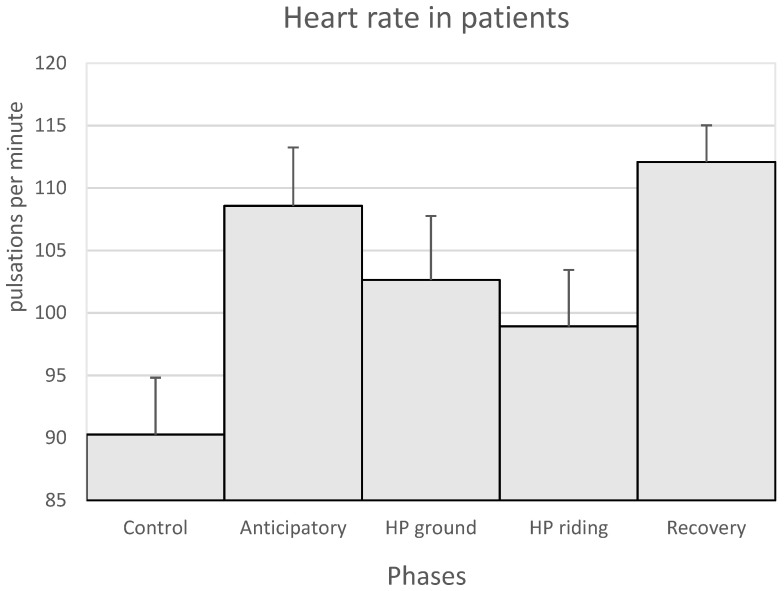
Mean values + SEM of the heart rate of the patients in the control and the anticipatory phases, the HP ground and HP riding phases (horse-patient interaction on the ground and on horseback, respectively) and the recovery phase. No significant differences were observed between the phases.

In patients, the HR was higher in the anticipatory and recovery phases (Figure 3). The highest T values were observed in the anticipatory phase (Table 6). The oxygen saturation of the patients in the anticipatory, HP ground and HP riding phases remained at slightly lower values than in the control phase. However, these values increased in the recovery phase. Regarding the sleep quality, a longer duration of deep sleep compared to light sleep was observed on the days of equine assisted therapy sessions versus control days (Table 6).

### 3.2. Changes of the Psychomotor and Emotional Parameters of the Patients after the Equine Assisted Therapy Sessions

#### 3.2.1. Therapists’ Assessment

The therapeutic team recorded a very positive evolution in the patients throughout this study according to the Bender rating scale [26]. Thus, at the motor level, the patients improved the coordination and the gross motor skills, such as gait, muscle tone, balance, head control, trunk control, etc. throughout the equine assisted therapy sessions. The patients also improved the fine motor skills such as the grip, hand support, etc. At a cognitive level, a progressive improvement of the attention, gaze maintenance, visual monitoring, communicative intention and perceptual ability of the patients was observed. At the affective-social level, an increased interest and interaction of the patients with people and animals around them was observed throughout the sessions. At the perceptual-sensitive level, the patients improved the general sensitivity, mainly at the proprioceptive and vestibular levels.

#### 3.2.2. Family Assessment Surveys

The Table 7 and Table 8 show the percentage of improvement of the patients and of the quality of life of the families, respectively, that was observed by the family members after the equine assisted therapy sessions. In addition to the aspects that appear in the cited tables, the following aspects were also perceived by the families: notable improvement of the overall development of the motor, visual and cognitive functions of the patients; improvement in knowledge and relationship of the patients with animals, as well as greater involvement and motivation towards the tasks. Likewise, the families observed that patients showed remarkable enthusiasm every time they attended the equine assisted therapy sessions. However, these data were not collected in the cited tables since the families expressed these aspects qualitatively, not quantitatively.

## 4. Discussion

### 4.1. Influence of Horse-Patient Interaction on Physiological and Behavioral Parameters in Horses and Patients during the Equine Assisted Therapy Sessions

The studies of the influence of horse-patient interaction in horses and patients during the equine assisted therapy sessions are still scarce and include, among others, studies on physiological, behavioral and hormonal parameters. In the present work we have measured different physiological and behavioral parameters in horses and patients throughout 9–10 weeks (1 session/week in each patient). According to several studies, physiological parameters are a good complement to the hormonal parameters and provide information of the balance of the vegetative (sympathetic/parasympathetic) Nervous System (NS) [19,20,27], since the values of the physiological parameters can provide information regarding the predominance of the sympathetic or parasympathetic NS.

In the present work, most of the physiological parameters of the horses and the patients were higher in the anticipatory phase than in the HP ground phase, which seems to show an anticipatory stress to the new event that the session entails, generating certain anxiety or excitement, due to the activation of the sympathetic NS (alert phase of the general syndrome of the stress adaptation) [28]. This would also explain the higher frequency of behavioral signs of stress that were observed in the horses in the anticipatory phase, which coincides with what was found by previous authors [29] in moments prior to the equine assisted therapy sessions.

In the HP ground phase, most of the physiological parameters decreased in the horses and in the patients, thus reflecting the relaxation of both (horses and patients) in the interaction phase on the ground, which seems to indicate a predominance of the parasympathetic NS, as has been suggested by other authors. Thus, other authors [30] studied the HR and the cortisol level in patients with intellectual disabilities and in the therapy horses. The parameters were measured at different moments of horse-patient interaction, observing a tendency to decrease both parameters in patients and horses during the cited interaction, which, according to the authors, reflects a decrease in stress by predominance of the parasympathetic NS. On the other hand, other studies evaluated the HR and the behavior in nine horses at moments of rest (control) and in different moments of the therapy sessions with patients that had physical and mental disabilities [20]. Their results showed that the therapy sessions did not significantly change the evaluated parameters in the horses. According to these authors, the changes that were detected in the horses seemed to be more related to curiosity than to stress. Similarly, in the present work, the behavioral parameters of stress that were observed in the horses after the anticipatory phase were mainly related to exogenous factors from the environment (food, noise, etc.), not observing a direct relationship of these signs with the interaction with the patient.

In the HP riding phase of the present work, the increase of the HR and R in horses corresponded to the physical activity of this phase, coinciding with what was observed by other authors [29] in equine assisted therapy sessions during the activity on the track. However, the evaluated parameters remained at low values in the patients during the HP riding phase (similar to those of the HP ground phase), which may correspond to the passive-active movement of the patients (with effortless advancement) that occurs during the back riding (passive equine assisted therapy), which helps the patients to stay relaxed during this phase.

In the recovery phase of the present work, the horse returned to the resting area and recovered their normal physiological values (similar to the control values), that also coincides with the results that were found by other authors [29] at the end of the equine assisted therapy sessions. However, in the patients, most of the parameters increased in the recovery phase, which seems to reflect a certain “reactivation” after the relaxation phase.

From what is above described it is observed that, on the whole, in the horses and in the patients there is an “alternation” of activation and relaxation phases, in which the horses respond more to external factors (activity, food, etc.) than to the own interaction with the patients, while the patients show a higher correlation of their parameters with the interaction with the horses, with higher relaxation in the interaction phases (HP ground and HP riding phases) and increased agitation in the anticipatory phase- and in the recovery phase. On the other hand, the improvement of the quality of sleep (sleeping phase) on the days of equine assisted therapy show that these sessions end up generating a good state of relaxation in the patients.

Our results coincide with those found by other authors. Thus, other studies measured HR, cortisol, and behavioral parameters in horses and patients with post-traumatic stress disorders and concluded that horse-patient interactions were just as stressful as the horses’ interactions with healthy people (control) [18]. These authors suggested that horses respond more to physical stimuli from humans, which increase horse curiosity and attention, than to emotional stimuli from humans. In 2018, other authors [19] performed five sessions of equine assisted therapy in military veterans with symptoms of post-traumatic stress disorders. The sessions were carried out on the ground, through brushing activities, walks, etc. HR, R and blood pressure of the veterans were measured in different moments of the sessions. In the therapy horses, HR and the levels of plasma cortisol and oxytocin were also measured. The evaluated parameters did not show differences in horses or veterans. However, a decrease in post-traumatic stress was observed in veterans after equine assisted therapy sessions.

Regarding the weekly evolution of the parameters, when comparing the HR values of the horses throughout the 9–10 weeks of the present study, a tendency to decrease these values was observed in the anticipatory phase. Similarly, a tendency to decrease the behavioral signs of stress of the horses in the anticipatory and recovery phases was observed throughout the experiment. These results seem to indicate a gradual adaptation of the horses to the sessions throughout the weeks. Similarly, other authors [19], when comparing HR between sessions, observed a gradual decrease in this parameter in horses in the first days.

In conclusion, the results found in the present study seem to indicate that the equine assisted therapy sessions do not produce any kind of stress in horses different from that produced by any activity or external stimulus. This fact, together with the well-being that the equine assisted therapy produces in the patients, justifies its use and shows its benefit as complementary therapy in therapeutic programs. However, more studies with a higher number of horses and patients are needed, as well as other studies that allow a classification based on the patient profile. Currently, as collected by other authors [4], the populations studied are heterogeneous and this can alter the results when mixing different pathologies. Likewise, it is necessary to complement the analysis of physiological and behavioral parameters with hormonal studies to establish a good correlation and a better understanding of the emotional response in horses and patients. These studies will also make it possible to objectify and quantify the efficacy of these therapies.

### 4.2. Influence of the Equine Assisted Therapy Sessions on the Psychomotor and Emotional Parameters of the Patients and on the Well-Being of the Families

Many studies show a positive effect of the equine assisted therapy in cerebral palsy and other pathologies that involve psychomotor disorders [1,2,3,4,5,6,7]. In 2016, a meta-analysis and systematic review on equine assisted therapy worldwide was carried out [5]. The aspects where the best results were found were balance, gross motor functions, sociability, affection, displacement, and symmetry. According to the authors of the cited study [5] the equine assisted therapy is a complementary method in the process of neurorehabilitation in the cerebral palsy, not only for its effects at a neuromotor level, but also for its benefits in the mental and behavioral condition of the patient, promoting learning and the motor functions.

Our results concur with those found by other authors, in such a way that the patients of this study showed an improvement of the gross motor skills (gait, balance, muscle tone, etc.) and fine motor skills (grip, coordination of small muscle movements), in addition to cognitive, perceptual and affective-social parameters. However, as indicated by other authors [4], the most significant problem for generalizing the results of these studies are the small sample sizes, as well as their heterogeneity in terms of age or clinical manifestations of the patients.

In relation to the quality of life of the families and of the patients, the results of the surveys show an improvement of the quality of life of the families, especially due to the benefits that they perceived in the locomotor aspects of the patients, together with an increase in their self-esteem, an improvement in the patient’s mood and an improvement of the bond of the patients with the families as well as of the interaction of the patients with other people around them, etc. These results coincide with those collected by previous authors [4,31], that observed that the patients improved their perception of themselves in parallel with the improvement of their motor development, which could facilitate the participation of children in society.

## 5. Conclusions

Most of the physiological parameters of the patients, as well as the physiological parameters and behavioral signs of stress of the horses increased in the anticipatory phase, probably due to the activation of the sympathetic nervous system that occurs during the alert phase of the general syndrome of the stress adaptation. On the contrary, these parameters decreased in patients and horses at the end of the HP ground phase, which indicates a relaxation probably associated with the activation of the parasympathetic nervous system. In the HP riding phase, the horses increased the HR and the R values, due to the physical activity, while the patients, given that they performed a passive-active movement during the back riding, maintained a state of relaxation, with low HR values. In the recovery phase, the horses relaxed, decreasing all their physiological parameters and most of the behavioral signs of stress, while the patients increased their HR values. On the other hand, the quality of patients’ sleep improved on the days they received the therapy sessions, showing a good final relaxation. After 9–10 sessions of equine assisted therapy, the patients improved their fine and gross motor functions, as well as the cognitive, emotional and affective-social parameters. These benefits improved the quality of life of the patients and of their families, according to the data collected in the surveys. These results, together with the fact that the therapy horses did not show a stress directly attributable to the therapy activities, but more related to external factors of the environment, allow us to conclude that the equine assisted therapy may be beneficial for patients and does not appear to affect the welfare of the therapy horses as long as the ethological characteristics of the horses are respected and their health is kept in good condition.

## Figures and Tables

**Table 1:** Characteristics of the therapy horses of this study.

Horse	Breed	A	Sex	W	H	TP	BL
1	PSB	20	M	400	1.51	1.77	1.50
2	PSB	17	F	450	1.55	1.94	1.53

PSB: pure Spanish breed; A: age (years); M: male; F: female; W: weight (kg); H: height at the withers (m); TP: thoracic perimeter (m); BL: body length or scapulo-ischium distance (from the scapulo-humeral joint to the area of greatest convexity of the gluteal muscles) (m).

**Table 2:** Characteristics of the patients of this study.

Patient	Diagnosis	A	Sex	W	H
1	General congenital hypotonia	2	M	10	0.80
2	Duchenne muscular dystrophy	9	M	38.5	1.22
3	Cerebral palsy	9	F	32	1.30

A: age (years); M: male; F: female; W: weight (kg), H: height (m).

**Table 3:** Phases of the equine assisted therapy sessions: duration, role of horses and patients, and data collection.

Phases	Anticipatory Phase	Interaction Patient-Horse on the Ground	Interaction Patient-Horse on Horseback	Recovery Phase
Duration	15 min	30 min	30 min	15 min
Role of the patients	The patients arrive at the therapy area. The therapy area is a covered area where conditions of safety and comfort are guaranteed for both horses and patients. In this phase, the therapy team explains to the patients the activities to be performed and prepare the material is going to be used during the session	Several activities that facilitate close contact between the patient and the horse were carried out in this phase, such as caressing and brushing, as well as several activities aimed at taking care of the horse’s basic needs, such as providing food and water. Other activities varied depending on the patient, but were mainly aimed at improving the following aspects: trunk control and body balance through brushing on unstable planes; fine motor skills through the performance of different horse hairstyles; oculo-manual coordination through manipulation of the horse’s clothing; the communicative through the interpretation of the horse’s signals and the elaboration of coherent responses to its demands; executive functions through planning, organization, decision-making and emotional management activities. These activities also served as preparation for riding phase, since these enhance the effect of assisted therapy on the horse.	The back riding technique was used in this phase. This technique is performed in such a way that the therapist sits behind the patient to provide support and align the patient. This technique improves the stability, the control and the postural hygiene. of the patient since the movements of the horse have a beneficial effect on these parameters.	After getting off the horse, the patients stay a little time in the center to recover from the activity.
Role of the horses	The horses are taken out of the box and taken to the therapy area. In this area the horses must have all their needs covered to guarantee a comfortable working climate, so they have water and forage on demand. To guarantee the safety of the patients and ensure a controlled session, the horses remain tied with a bridle and branch, but with sufficient head opening and branch distance, so that they can forage.	Horses interact with the patients, responding to the stimuli they receive from the care activities and any other activity specifically programmed by the therapeutic team for each patient.	The horse is guided towards the working track, either by the patient (accompanied by the therapist), or by the horse-trained guide. The patients access a platform placed close to the horse, through a ramp or ladder. From the platform, the patients easily get on the horses. The horses were mainly ridden at the walk in the track. Horses were led around the track with the help of a horse-trained guide.	The horses finish the activity on the track and return to their resting area, where they can rest and access food and some water. Thus, they can recover from the activity.
Data collection	At the beginning of this phase	At the end of this phase	At the end of this phase.	At the end of this phase

**Table 4:** Mean values ± SEM of the blood pressure and the temperature that were measured in the horses during the control and the anticipatory phases, HP ground and HP riding phases (horse-patient interaction on the ground and on horseback, respectively) and the recovery phase.

Physiological Parameters	Phases				
	Control	Anticipatory	HP Ground	HP Riding	Recovery
SBP	75.27 ^a^ ± 1.67	110.19 ^b^ ± 5.31	100.96 ^bc^ ± 3.16	92.59 ^ac^ ± 3.53	88.56 ^ac^ ± 3.52
DBP	48.40 ^a^ ± 3.31	66.52 ^b^ ± 4.73	60.77 ^ab^ ± 2.14	56.93 ^ab^ ± 2.25	53.78 ^a^ ± 2.05
T	37.27 ^b^ ±0.11	36.84 ^a^ ± 0.06	36.88 ^a^ ± 0.04	36.88 ^a^ ± 0.05	36.77 ^a^ ± 0.06

Parameters: SBP and DBP: systolic and diastolic blood pressures, respectively (mm Hg). T: temperature (°C). Different superscripts within each row indicate significant differences between the phases (*p* < 0.05), for each parameter.

**Table 5:** Mean values (%) ± SEM of the behavioral parameters that are related to stress in the horses in the anticipatory phase, the HP ground and HP riding phases (horse-patient interaction on the ground and on horseback, respectively) and the recovery phase.

Behavioral Signs	Phases			
	Anticipatory	HP Ground	HP Riding	Recovery
Change of postural expression	6.73 ^a^ ± 3.27	2.88 ^a^ ± 2.11	1.92 ^a^ ± 1.92	5.77 ^a^ ± 4
Kick the ground	38.46 ^a^ ± 3.98	10.58 ^b^ ± 3.71	0 ^b^ ± 0	14.42 ^b^ ± 5.91
Head lowered	18.27 ^a^ ± 2.21	1.92 ^b^ ± 1.33	0 ^b^ ± 0	0.96 ^b^ ± 0.96
Ears back	3.26 ^a^ ± 1.79	1.09 ^a^ ± 1.09	2.17 ^a^ ± 1.50	1.09 ^a^ ± 1.09
Ears in a listening attitude	23.08 ^a^ ± 2.37	15.38 ^ac^ ± 2.8	30.77 ^b^ ± 2.88	7.69 ^c^ ± 3.33
Gentle head shake	1.92 ^a^ ± 1.33	0.96 ^a^ ± 0.96	0.96 ^a^ ± 0.96	0.96 ^a^ ± 0.96

The values are expressed in terms of percentage, that is, the frequency of appearance of each sign in each phase. Different superscripts within each row indicate significant differences between the phases (*p* < 0.05) for each parameter.

**Table 6:** Mean values ± SEM of some physiological parameters the patients during the control and the anticipatory phases, the HP ground and HP riding phases (horse-patient interaction on the ground and on horseback, respectively) and the recovery phase. The sleeping phase refers to the time (expressed in minutes) of deep and light sleep on equine assisted therapy days.

Physiological Parameters	Phases					
	Control	Anticipatory	HP Ground	HP Riding	Recovery	Sleeping
O	97.36 ^a^ ± 0.45	94.39 ^a^ ± 1.23	94.86 ^a^ ±1.14	95.92 ^a^ ± 0.83	97.92 ^a^ ± 0.26	-
T	35.84 ^a^ ± 0.26	36.26 ^b^ ± 0.05	36.18 ^ab^ ± 0.06	36.20 ^ab^ ± 0.03	36.18 ^ab^ ± 0.07	
LS	457 ^a^ ± 47.7	-	-	-	-	496.7 ^a^ ± 23.5
DS	95.7 ^a^ ± 10.4	-	-	-	-	120.3 ^a^ ± 14.5

Parameters: O: oxygen saturation (%); T: temperature (°C); LS and DS: light and deep sleep (minutes). Different superscripts within each row indicate significant differences (*p* < 0.05) between the phases, for each parameter.

**Table 7:** Mean values ± SEM of improvement percentage of different parameters of the patients. These parameters were collected from the surveys that were filled out by the families of the patients after 9–10 sessions of equine assisted therapy.

Influence of the Equine Assisted Therapy on the Improvement of Different Parameters of the Patients, According to the Surveys.												
S	I	A	T	C	G	FI	NFI	M	E	IM	AC	SE
30 ± 30	30 ± 30	40 ± 40	100 ± 0	100 ± 0	100 ± 0	33.33 ± 17.64	70 ± 30	53.33 ± 6.67	46.67 ± 24.04	53.33 ± 26.67	46.67 ± 24.04	33.33 ± 17.64

The values are expressed as the percentage of improvement that was observed by the families in each item. Sleep quality (S); intestinal transit (I); appetite (A), trunk control (T); cephalic control (C); gait control (G), bonding and interaction with the family (FI); interaction with people who do not belong to the family (NFI); improvement of mood (M); improvement of emotional regulation (E); illusion-motivation for carrying out activities, that is, degree of involvement of patients in the tasks and activities that are proposed (IM); attention-concentration (AC); self -esteem(SE).

**Table 8:** Mean values ± SEM of improvement percentage of different parameters related to the quality of life of the families. These parameters were collected from the surveys that were filled out by the families of the patients after 9–10 sessions of equine assisted therapy.

Influence of Equine Assisted Therapy on the Quality of Life of the Families						
s	exp	if	nif	ns	em	p
66.67 ± 33.33	66.67 ± 33.33	66.67 ± 33.33	86.67 ± 13.33	66.67 ± 33.33	66.67 ± 33.33	66.67± 33.33

Values are expressed as the percentage of improvement that was observed by the families in each item. Social act (s); way of sharing experiences with other families (exp); improvement of intra-family relationships (if) and with people outside the family (nif); help to normalize and accept the situation (ns); release of tension (emotional discharge) (em) and improvement of the perception of the situation (p).

## Data Availability

The data does not come from any database.

## Supplementary Materials

The following are available online at https://www.mdpi.com/article/10.3390/ani11123527/s1, Table S1: Motor, cognitive and perceptual-sensitive parameters that were evaluated in the patients.
