# Supplementary material for: Pilot Study of the Influence of Equine Assisted Therapy on Physiological and Behavioral Parameters Related to Welfare of Horses and Patients
Source: Animals (Basel). 2021 Dec 10;11(12):3527. doi: 10.3390/ani11123527 (PMC8698107; doi:10.3390/ani11123527)
Supplement: Supplementary file 1 [file animals-11-03527-s001.zip › animals-1446683-supplementary.pdf]

Table S1 Motor, cognitive and perceptual-sensitive parameters that were evaluated in the patients.

**MOTOR AREA**			
**Gross motor parameters**			
Standing		Habitual commuting at home	Crawling / Crawling-Rolling / Walking / Wheelchair
Sitting			
March		Usual displacements over long distances	Walking / Wheelchair / Stroller
Head control		Transition to horse	Dependent / Supported / Self-employed
Trunk control	Yes / No / With support	Needs back riding	Yes / No / Depends on activity
Jump		Predominant trunk pattern	Flexor / Extensor / Left / Right
Monopodal support		Has muscle weakness	Anterior muscle chain / Posterior muscle chain / Specific
Up / down ramp		Pelvis position in lateral plane	Retroversion / Anteversion / Mobile
Up / down stairs		Dissociation between waists	Yes / On horseback riding
Kneeling position		Quadruped position	Yes / No / With support
Knight position			
**Muscular tone**			
Dystonia	Observations of the tone of the muscles of the locomotor system: increased, decreased, etc.		
**Fine motor parameters**			
Grip	Yes / No /	Hand support	Adequate / Limited
Midline crossing	Adequate / Limited		
**Coordination**			
Eye-hand-mouth			
Bimanual	Adequate / Limited		
Ocular manual			
**COGNITIVE AREA**			
**Attention**			
Selective	Adequate / Inadequate / Depends on the activity	Sustained	Adequate / Inadequate / Depends on the activity
Divided		Processing speed	
**Memory**			
Short term		Retrograde amnesia	
Long term	Altered / Unaltered	Anterograde amnesia	Yes / No
**Executive functions**			
Working memory		Decision making	
Task planning		Temporal estimate	Altered / Unaltered

Reasoning	Altered / Unaltered	Body self-knowledge	
Flexibility		Dual execution	
Inhibition		Simultaneous execution	
**Symbolic game**			
Appears / does not appear			
**Emotional development**			
Recognize and feel basic emotions	Yes / No	Self-control problems	Yes / No
Self-concept	Adequate / Inadequate	Behavior problems	
**Communication**			
Communicative intention	Yes / No	Anticipation capacity	Yes / No
Eye contact		Verbal communication	
Verbal imitation		Non-verbal communication	Characteristics: babbling, guttural sounds, etc.
Simple commands	Adequate or inadequate understanding and execution		
**PERCEPTUAL-SENSITIVE AREA**			
Vision	Hypersensitive / Hyposensitive		
Hearing			
Smell			
Touch			
Taste			
Proprioceptive			
Vestibular			
